# Antibacterial Effect of Chitosan-Modified Fe_3_O_4_ Nanozymes on *Acinetobacter baumannii*

**DOI:** 10.4014/jmb.2107.07046

**Published:** 2021-10-19

**Authors:** Wang Wenjun, Wu ziman, Shi peiru, Wu pinyun, Qin peng, Yu lin

**Affiliations:** 1 The First Affiliated Hospital of Guangzhou Medical University, Guangzhou, Guangdong 510120, P.R. China; 2 Guangzhou Medical University (KingMed school of Laboratory Medicine), Guangzhou, Guangdong 510182, P.R. China

**Keywords:** Nanozyme, antibacterial activity, *Acinetobacter baumannii*

## Abstract

The aim of this study was to determine whether the antibacterial activity of chitosan-modified Fe_3_O_4_ (CS@Fe_3_O_4_) nanomaterials against *Acinetobacter baumannii* (*A. baumannii*) is mediated through changes in biofilm formation and reactive oxygen species (ROS) production. For this purpose, the broth dilution method was used to examine the effect of CS@Fe_3_O_4_ nanoparticles on bacterial growth. The effects of CS@Fe_3_O_4_ nanoparticles on biofilm formation were measured using a semi-quantitative crystal violet staining assay. In addition, a bacterial ROS detection kit was used to detect the production of ROS in bacteria. The results showed that CS@Fe_3_O_4_ nanoparticles had a significant inhibitory effect on the colony growth and biofilm formation of drug-resistant *A. baumannii* (*p* < 0.05). The ROS stress assay revealed significantly higher ROS levels in *A. baumannii* subjected to CS@Fe_3_O_4_ nanoparticle treatment than the control group (*p* < 0.05). Thus, we demonstrated for the first time that CS@Fe_3_O_4_ nanoparticles had an inhibitory effect on *A. baumannii* in vitro, and that the antibacterial effect of CS@Fe_3_O_4_ nanoparticles on drug-resistant *A. baumannii* was more significant than on drug-sensitive bacteria. Our findings suggest that the antibacterial mechanism of CS@Fe_3_O_4_ nanoparticles is mediated through inhibition of biofilm formation in drug-resistant bacteria, as well as stimulation of *A. baumannii* to produce ROS. In summary, our data indicate that CS@Fe_3_O_4_ nanoparticles could be used to treat infections caused by drug-resistant *A. baumannii*.

## Introduction

Nanozymes, nanomaterials with enzyme-like catalytic activity, are a new generation of artificial enzyme that can catalyze the substrates of biological enzymes under near physiological conditions. Their catalytic behavior, reaction kinetics and catalytic mechanisms are similar to those of natural enzymes. In 2019, Jiao *et al*. [1] at the Chinese Academy of Sciences first reported that Fe_3_O_4_ magnetic nanoparticles exhibited mimetic enzyme activities similar to those of natural peroxidases. Since then, more than 300 types of nanomaterials have been found to have enzymatic activity. Compared with natural enzymes and traditional mimic enzymes, nanozymes have high catalytic efficiency, multiple functions, good stability, low cost and easy large-scale preparation [2]. In the biomedical field, nanozymes can be used in clinical detection, tumor diagnosis and treatment, cell protection, anti-aging, and other important areas related to human health [3].

The Fe_3_O_4_ nanozyme is a typical metal oxide nanozyme. Fe_3_O_4_ nanoenzymes have the same catalytic characteristics as horseradish peroxidase (HRP), and can catalyze H_2_O_2_ to destroy biofilm matrices [5]. However, due to their toxic effect on cells, subsequent studies have developed various modification methods to reduce their cytotoxicity. Chitosan nanoparticles can be applied to deliver antimicrobial drugs, which further enhances the efficiency and stability of the antimicrobial agent [6]. The proposed antibacterial mechanism of chitosan under acidic conditions involves the binding of the negatively charged bacteria to the protonated amino group on the chitosan molecular chain, leading to disruption of the cell, and subsequent inhibition of bacterial growth and reproduction [4]. Tian *et al*. [7] demonstrated that the chitosan-modified Fe_3_O_4_ (CS@Fe_3_O_4_) nanozyme had lower cytotoxicity than the sodium oleate-modified Fe_3_O_4_ nanozyme, thereby leading to the clinical application of CS@Fe_3_O_4_ nanozymes.

*Acinetobacter baumannii* (*A. baumannii*) is a gram-negative bacilli mainly associated with community-acquired pneumonia and hospital infection. *A. baumannii* can secrete many virulence factors, which are related to its pathogenicity and drug resistance [8]. In addition, the extracellular matrix secreted by *A. baumannii* can adhere to the body, and most *A. baumannii* can form biofilms, which further enhance its pathogenicity [9]. *A. baumannii* infection leads to prolonged disease and high mortality. In addition, most *A. baumannii* strains have now developed resistance to a variety of drugs, including quinolones, aminoglycosides, broad-spectrum cephalosporins and other common clinical antibiotics [10]. In 2014, China's Ministry of Health issued technical guidelines for the prevention and control of hospital infections caused by multidrug-resistant bacteria, clearly defining multidrug-resistant bacteria as bacteria that are resistant to three or more types of antibiotics in clinical use at the same time. In recent years, the increasing occurrence of multidrug-resistant *A. baumannii* has had a serious impact on human health. Indeed, *A. baumannii* has become one of the most intractable pathogens in global healthcare institutions [11].

Here, CS@Fe_3_O_4_ nanozymes were used in in vitro antibacterial experiments to determine their effects on *A. baumannii* biofilms, as well as their impact on reactive oxygen species (ROS) levels in *A. baumannii*.

## Materials and Methods

### Bacteriostasis Assay Using the Broth Dilution Method

A 500 μg/ml working solution of CS@Fe_3_O_4_ nanoparticles (Ruixi Biotechnology, China) was prepared in double-distilled water. An *A. baumannii* colony was diluted in 4 ml Mueller-Hinton broth medium to prepare a bacterial suspension working solution (1 × 10^7^ CFU/ml, OD 600 nm = 0.06) (Huankai Microbial Technology, China). The CS@Fe_3_O_4_ nanoparticles were added to the bacterial suspension (final concentration of 125 μg/ml), and the final working solution was placed in 96-well plates and cultured for 24 h. In the control group, double-distilled water replaced the CS@Fe_3_O_4_ nanoparticles.

### Semi-Quantitative Crystal Violet Staining Assay

Biofilm formation was measured after 24 and 48 h, as described previously [12, 13]. An *A. baumannii* suspension (1 × 10^7^ CFU/ml, OD 600 nm = 0.06) was prepared. CS@Fe_3_O_4_ nanoparticles were diluted to a concentration of 500 μg/ml. In the experimental group, the CS@Fe_3_O_4_ nanoparticle working solution (50 μl) and bacterial suspension (150 μl) were added to 96-well plates. In the control group, the CS@Fe_3_O_4_ nanoparticle working solution was replaced with double-distilled water. After incubation for 24 h, the culture medium was removed, and each well was washed three times with PBS. The biofilms were then fixed with methanol (200 μl) for 15 min, air-dried, and stained with 2% crystal violet solution (200 μl) for 5 min at room temperature. After the excess stain was removed, samples were washed three times with PBS and air-dried at room temperature. Adherent crystal violet was dissolved in 95% alcohol (200 μl) and the plates were shaken for 20 min. The optical density (OD) was measured at 570 nm using a microplate reader (Biotek, USA).

### Microscopic Analysis of Crystal Violet Staining

CS@Fe_3_O_4_ nanoparticle working solution (125 μl) and bacterial suspension (375 μl) were added to 24-well plates containing a clean sterile glass cover slip. In the control group, the CS@Fe_3_O_4_ nanoparticle working solution was replaced with double-distilled water. After incubation for 24 and 48 h, the glass cover slips were gently rinsed with PBS to remove non-adherent bacteria and then stained with 2% crystal violet for 30 min. Slides were washed with water to remove excess crystal violet and then visualized using an Olympus CKX41 microscope (Japan).

### ROS Stress Test

The ROS stress test was carried out as described previously [14] using a bacterial ROS fluorescence detection kit (Beibo Biotechnology, China). The experimental group was treated with a CS@Fe_3_O_4_ nanoparticle working solution (500 μg/ml) prepared using double-distilled water. The control group was treated with double-distilled water (200 μl). The fluorescence intensity (excitation light was 488 nm, emission light was 530 nm) was measured with a fluorescence enzyme reader (Biotek). After the absorbance had been measured, the bacterial solution was centrifuged at 12,000 ×*g* for 20 min, then placed on a glass slide and observed using an Olympus fluorescence microscope BX43.

### Statistical Methods

The paired *t*-test was used to analyze the biofilm inhibition and ROS stimulation data. The Wilcoxon nonparametric test was used to analyze the 24 h data. All statistical analyses were performed using SPSS version 20.0 (IBM), and all figures were generated using GraphPad Prism 5.01 (GraphPad Software). *p* < 0.05 was considered statistically significant.

## Results

### CS@Fe_3_O_4_ Nanoparticles Inhibit *A. baumannii*

**Fig. 1:**
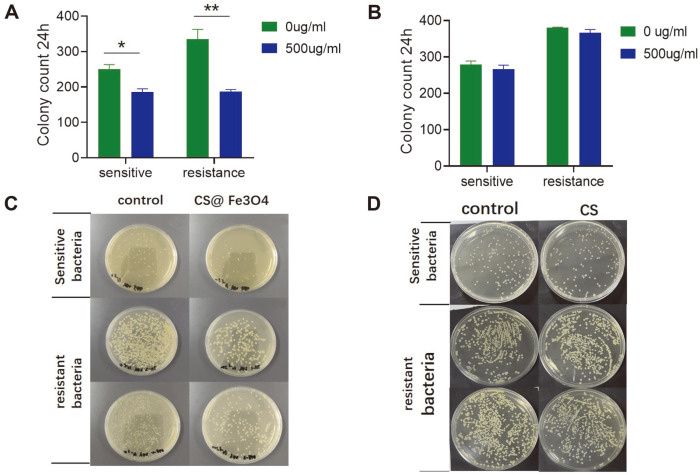
The inhibition rate of CS@Fe_3_O_4_ nanoparticles (**A**) and chitosan (**B**) on *A. baumannii* was detected by the colony formation assay. Data are shown as the mean ± SD of three independent experiments. *p < 0.05, **p < 0.01. The broth dilution method was used to determine the inhibition rate of CS@Fe_3_O_4_ nanoparticles (**C**) and chitosan (**D**) on *A. baumannii*.

Based on our drug sensitivity test, drug-sensitive and drug-resistant bacteria were selected (Table S1). Next, we performed in vitro bacteriostatic experiments and found that CS@Fe_3_O_4_ nanoparticles had a significant inhibitory effect on *A. baumannii* compared with the control group in both drug-sensitive (*p* < 0.05) and drug-resistant (*p* < 0.01) bacteria (Fig. 1A). The number of colonies growing in the experimental group was significantly less than in the control group (Fig. 1C). Importantly, chitosan alone (at the same concentration) had no antibacterial effect on the number of colonies. (*p* > 0.05) (Fig. 1B), It also had no effect on the colony formation of *A. baumannii* (Fig. 1D).

### CS@Fe_3_O_4_ Nanoparticles Inhibit the Biofilm Formation of Drug-Resistant *A. baumannii*

**Fig. 2:**
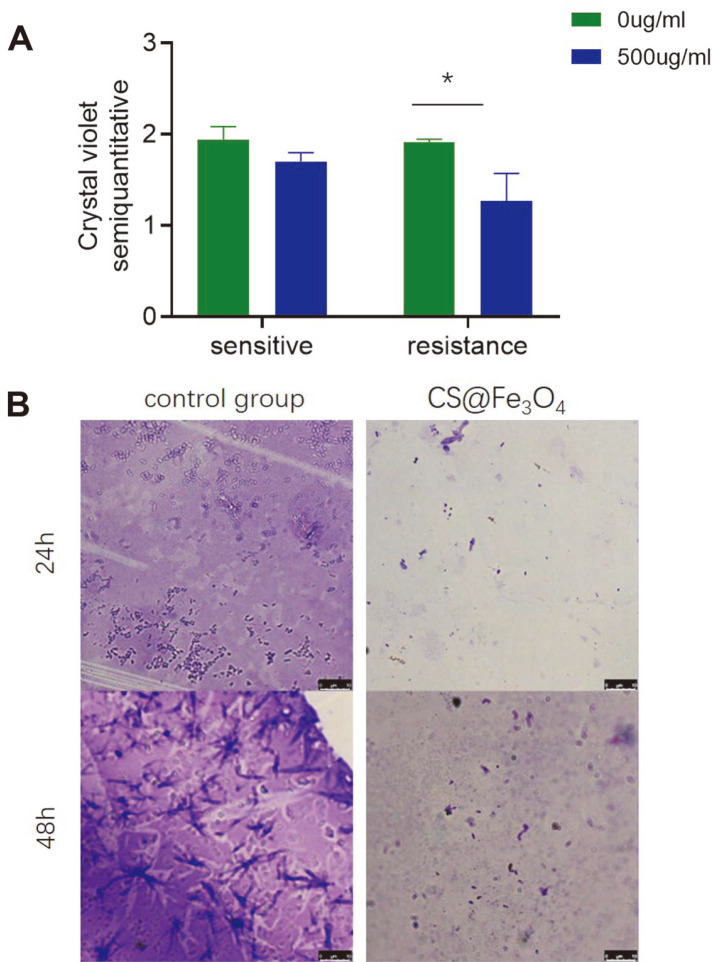
A. The semi-quantitative crystal violet staining assay was used to determine the amount of bacterial biofilm. OD values are given as the mean ± SD of three independent experiments. * *p* < 0.05. B. Microscopic examination of the effects of CS@Fe_3_O_4_ nanoparticles on biofilm formation were observed by crystal violet staining after 24 and 48 h.

The crystal violet quantitative assay revealed that CS@Fe_3_O_4_ nanoparticles had an inhibitory effect on the biofilm formation of drug-resistant *A. baumannii* (*p* < 0.05), but not drug-sensitive bacteria (Fig. 2A). Microscopic examination of the biofilms revealed that the biofilm of the control group was large, dense and dark purple, whereas the biofilm of the experimental group was significantly smaller with light purple staining (Fig. 2B).

### CS@Fe_3_O_4_ Nanoparticles Stimulate *A. baumannii* to Produce ROS

**Fig. 3:**
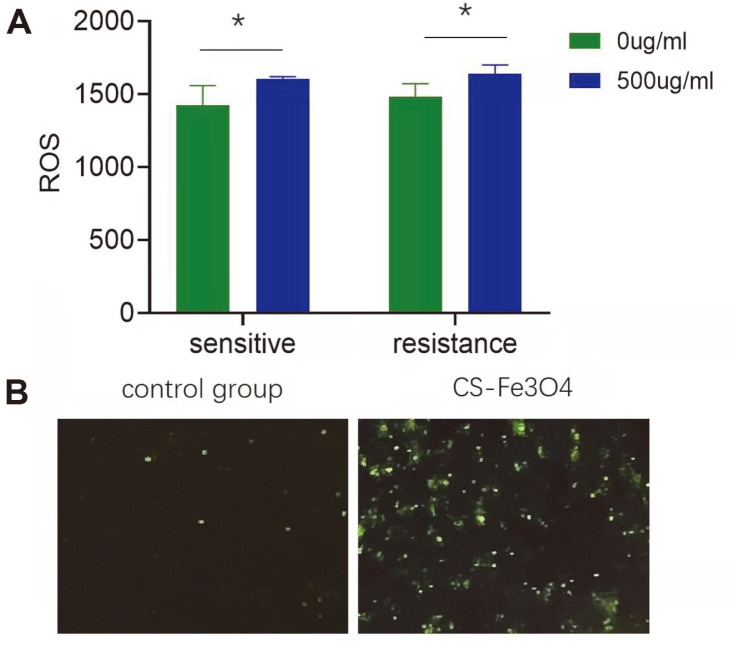
A. Quantitative analysis of the effects of CS@Fe_3_O_4_ nanoparticles on bacterial oxidative stressactivated oxygen content were determined using a commercially available bacterial ROS fluorescence detection kit. Data are shown as the mean ± SD of three independent experiments. * *p* < 0.05. B. Examination of the effects of CS@Fe_3_O_4_ nanoparticles on ROS levels in *A. baumannii* by fluorescent microscopy.

A bacterial ROS kit was used to quantify ROS production in the bacteria. ROS levels were significantly increased in the experimental group for both drug-sensitive and drug-resistant (*p* < 0.05) bacteria (Fig. 3A). Microscopic examination revealed an increase in both the number of ROS-positive bacteria, as well as the staining intensity in the experimental group compared to the control group (Fig. 3B).

## Discussion

*A. baumannii* has become one of the main sources of infection in intensive care unit patients, and a large number of strains are now resistant to common antibiotics [15]. Thus, the development of a new drug for multidrug-resistant *A. baumannii* is critical. One of the pathogenic properties of *A. baumannii* is the production of a large amount of biofilm. Bacterial biofilms have a protective effect on bacteria, and thus, inhibition of biofilm function or formation could reduce bacterial resistance [16]. Many chronic wound infections have been shown to be associated with biofilms [17]. *A. baumannii* can form biofilms that attach to the surface of ventilators and tracheal intubations [18], and can also occur in the respiratory tract and skin of patients [19]. The formation of biofilms not only enhances the resistance of bacteria to antibiotics [20], but also stimulates the host immune system to release a large number of cytokines to trigger an immune response.

Fe_3_O_4_ nanozymes exhibit triple enzyme-like activities including peroxidase, catalase, and superoxide dismutase [21], and are a typical metal oxide nanoenzyme that exhibits catalytic properties similar to HRP. Their catalytic activity is associated not only with the pH, reaction temperature and H_2_O_2_ concentration of the solution, but also with the nanoparticle size, with smaller particles leading to higher catalytic activity [22]. The Fe_3_O_4_ nanozyme was shown to catalyze H_2_O_2_ to destroy the biofilm matrix of *Pseudomonas aeruginosa*, and its bactericidal effect was more than 10 times higher than that of using H_2_O_2_ alone [23]. Recently, iron sulfide and Fe_3_O_4_ nanozymes were shown to not only destroy the biofilm formed by *Salmonella typhimurium*, but also prevent its formation [24]. In addition, nanomaterials based on cerium ions have been shown to inhibit biofilm formation [25].

ROS are an important component of the immune response, and play a crucial role in eliminating invading pathogens, as well as promoting oxidative stress and damaging cellular proteins and lipids [26]. Mammalian macrophages and neutrophils can directly internalize foreign pathogens and degrade them in lysosomes in a ROS-dependent manner. Many lysosomal enzymes catalyze the production of ROS in an acidic environment, leading to the inactivation of biological macromolecules such as nucleic acids and proteins [27]. Nanozymes have the ability to regulate ROS levels [21, 28], which may account for the antibacterial activity of nanozymes [29]. Here, we found that CS@Fe_3_O_4_ nanoparticles can stimulate ROS production in both drug-sensitive and drug-resistant *A. baumannii*, disrupting internal metabolism and exerting an antibacterial role.

We found that CS@Fe_3_O_4_ nanoparticles had a more significant inhibitory effect on drug-resistant bacteria than drug-sensitive bacteria. Thus, we next sought to determine the mechanisms mediating the antibacterial effect of CS@Fe_3_O_4_ nanoparticles. We found that CS@Fe_3_O_4_ nanoparticles could inhibit the formation and function of drug-resistant *A. baumannii* biofilms, and significantly increase the ROS content in the drug-resistant *A. baumannii*. Furthermore, we found that in drug-sensitive *A. baumannii*, CS@Fe_3_O_4_ nanoparticles stimulated the production of ROS, but had no significant inhibitory effects on the biofilm. These findings may explain the differential antibacterial effects of CS@Fe_3_O_4_ nanoparticles on drug-sensitive and drug-resistant *A. baumannii*, and highlight the need to develop new antibiotics for the treatment of drug-resistant bacteria [30].

In conclusion, our study is the first description of the antibacterial effects of CS@Fe_3_O_4_ nanoparticles on drug-resistant and drug-sensitive strains of *A. baumannii*. In addition, the antibacterial mechanism of CS@Fe_3_O_4_ nanoparticles was preliminarily explored. Future studies will examine the molecular pathways and targets within the bacteria. This study provides a solid foundation for the development of antimicrobial agents to treat drug-resistant *A. baumannii*.

## Supplemental Materials

Supplementary data for this paper are available on-line only at http://jmb.or.kr.
