# Supplementary material for: Antibacterial Effect of Chitosan-Modified Fe3O4 Nanozymes on Acinetobacter baumannii
Source: J Microbiol Biotechnol. 2021 Oct 19;32(2):263–7. doi: 10.4014/jmb.2107.07046 (PMC9628855; doi:10.4014/jmb.2107.07046)
Supplement: Supplementary file 1 [file jmb-32-2-263-supple.pdf]

J. Microbiol. Biotechnol.
https://doi.org/10.4014/jmb.2107.07046

Supplementary Materials

Materials and methods - Drug Sensitivity Test

The paper diffusion method (K-B method) was used for drug sensitivity test, and the results were recorded and analyzed. The 10 kinds of antibiotics commonly used by ab bacteria include aminoglycosides, broad-spectrum cephalosporins, folate metabolism inhibitors, carbapenems, antibiotics, Simple ring β- Lactams, Tetracyclines, penicillins+ β- Lactamase inhibitors, fluoroquinolones, phosphonates and polymyxin. Sensitive AB bacteria are sensitive to 10 kinds of commonly used antibiotics. Multidrug resistant Acinetobacter baumannii (MDRAB): Acinetobacter baumannii resistant to 3 or more of 10 kinds of antibiotics. The antibiotics include (abbreviate) :Minocycline (mn) , Meropenem antibiotics (mem),Ceftazidime(taz), Cefoperazone/sulbactam(sfp), Amikacin(an), Compound sulfamethoxazole(sxt), Cegepime(fep), Imipenem(imi), Ciprofloxacin(cip), Piperacillin/tazobactam(tzp), Tigecycline(tgc), Levofloxacin(lev), Tobramycin(tob), Doxycycline(dx), Colistin(cs) , Ticarcillin/ Clavulanic acid(tcc).

Results of drug sensitivity test

A total of 30 strains of AB bacteria were randomly collected from the Microbiology Department of the First Affiliated Hospital of Guangzhou Medical University, including 10 sensitive bacteria and 20 multidrug-resistant bacteria. The results of drug sensitivity of 20 MDRAB are shown in Table 1.

Table 1 Summary of drug sensitivity results of 20 strains of MDRAB

antibiotics	S<br>N(N%)	I<br>N(N%)	R<br>N(N%)
mn	9(45.0)	7(35.0)	4（20.0）
mem	0(0.0)	0(0.0)	20（100.0）
taz	0(0.0)	0(0.0)	20（100.0）
sfp	1(5.0)	1（5.0）	18（90.0）
an	2(10.0)	0(0.0)	18（90.0）
sxt	8(40.0)	0(0.0)	12（60.0）
fep	7(35.0)	5（25.0）	8（40.0）
imi	0(0.0)	0(0.0)	20（100.0）
cip	0(0.0)	0(0.0)	20（100.0）
tzp	0(0.0)	0(0.0)	20（100.0）
tgc	19(95.0)	1（5.0）	0(0.0)
lev	3(15.0)	1（5.0）	16（80.0）
tob	5(25.0)	0(0.0)	15（75.0）
dx	1(5.0)	0(0.0)	19（95.0）
cs	1(5.0)	19（95.0）	0(0.0)
tcc	0(0.0)	0(0.0)	20（100.0）
